# Temperature responses of mutation rate and mutational spectrum in an *Escherichia coli* strain and the correlation with metabolic rate

**DOI:** 10.1186/s12862-018-1252-8

**Published:** 2018-08-29

**Authors:** Xiao-Lin Chu, Bo-Wen Zhang, Quan-Guo Zhang, Bi-Ru Zhu, Kui Lin, Da-Yong Zhang

**Affiliations:** State Key Laboratory of Earth Surface Processes and Resource Ecology and MOE Key Laboratory for Biodiversity Science and Ecological Engineering, Beijing Normal University, Beijing, 100875 People’s Republic of China

**Keywords:** Evolutionary speed hypothesis, Molecular evolution, Mutation accumulation, Mutation rate, Mutational spectrum, Oxidative DNA damage

## Abstract

**Background:**

Temperature is a major determinant of spontaneous mutation, but the precise mode, and the underlying mechanisms, of the temperature influences remain less clear. Here we used a mutation accumulation approach combined with whole-genome sequencing to investigate the temperature dependence of spontaneous mutation in an *Escherichia coli* strain. Experiments were performed under aerobic conditions at 25, 28 and 37 °C, three temperatures that were non-stressful for the bacterium but caused significantly different bacterial growth rates.

**Results:**

Mutation rate did not differ between 25 and 28 °C, but was higher at 37 °C. Detailed analyses of the molecular spectrum of mutations were performed; and a particularly interesting finding is that higher temperature led to a bias of mutation to coding, relative to noncoding, DNA. Furthermore, the temperature response of mutation rate was extremely similar to that of metabolic rate, consistent with an idea that metabolic rate predicts mutation rate.

**Conclusions:**

Temperature affects mutation rate and the types of mutation supply, both being crucial for the opportunity of natural selection. Our results help understand how temperature drives evolutionary speed of organisms and thus the global patterns of biodiversity. This study also lend support to the metabolic theory of ecology for linking metabolic rate and molecular evolution rate.

**Electronic supplementary material:**

The online version of this article (10.1186/s12862-018-1252-8) contains supplementary material, which is available to authorized users.

## Background

Temperature has long been recognized as a major determinant of mutation rate since H. J. Muller’s seminal work on mutation properties [1]. A positive temperature dependence is expected, as higher temperatures may increase both replication errors and DNA damage from free radicals produced as by-products of metabolism [2–6]. The temperature effect on spontaneous mutation is not only interesting on its own, but also crucial for our understanding of the biodiversity patterns on Earth, especially the latitudinal diversity gradient recognized since A. R. Wallace; this is because mutation is the ultimate source of genetic variation, the raw material for evolution and the emergence of biodiversity [7–13]. The evolutionary speed hypothesis (ESH) has well explained the greater speciation rates in the tropics, that is, warmer temperatures can lead to faster evolutionary processes due to shorter generation times, increased mutation rates and accelerated natural selection [14–17]. Recent theoretical research has also attempted to establish a neat relationship between molecular evolution rate and temperature and body size, based on an assumption that metabolic rate determines mutation rate [18–22].

Experimental studies conducted with a wide range of organisms including viruses, bacteria, eukaryotic microbes, plants and insects reached a consensus that temperatures that are extremely high and stressful for study organisms usually cause mutagenic effects (such high temperatures often result in very low growth rate or survival rate) [2, 23–25]. However, mixed results were found for temperature ranges that are benign and less-stressful for study organisms; for example, for a single species *Escherichia coli*, both positive and neutral temperature effects on mutation rate were reported for the temperature range 15–37 °C [26–28]. It is unclear whether the inconsistency between theoretical expectation and empirical evidence was due to the technical limitations of the experimental studies. In particular, the earlier experimental studies used the fluctuation test approach to estimate rates of mutations at certain reporter loci with obvious phenotypic consequences such as resistance to antibiotics or bacteriophages, which were unlikely to reflect the whole-genome property [29–31].

The present study has two aims. First, we would investigate the temperature effects on mutation rate and mutational spectrum in an *Escherichia coli* strain, using a mutation accumulation (MA) strategy combined with whole-genome sequencing (WGS), whereby major methodological limitations inherent in previous experimental studies could be overcome [30, 32]. An MA experiment involves establishing clonal populations from a single founder and then propagating each population with repeated single-individual bottlenecks. Due to the extremely low effective population size, fixation of all but strongly deleterious mutations proceeds in a nearly neutral fashion [33–37]. WGS allows identification of all mutations fixed during the MA procedure, and thus estimation of temperature dependences of both the rate and molecular spectrum of mutations [31]. Second, we investigate whether or not mutation rate and metabolic rate show a congruent temperature dependence, a prediction from the metabolic theory of ecology with an assumption of a constant mutation rate per mass-specific metabolic energy [19].

## Results

**Fig. 1:**
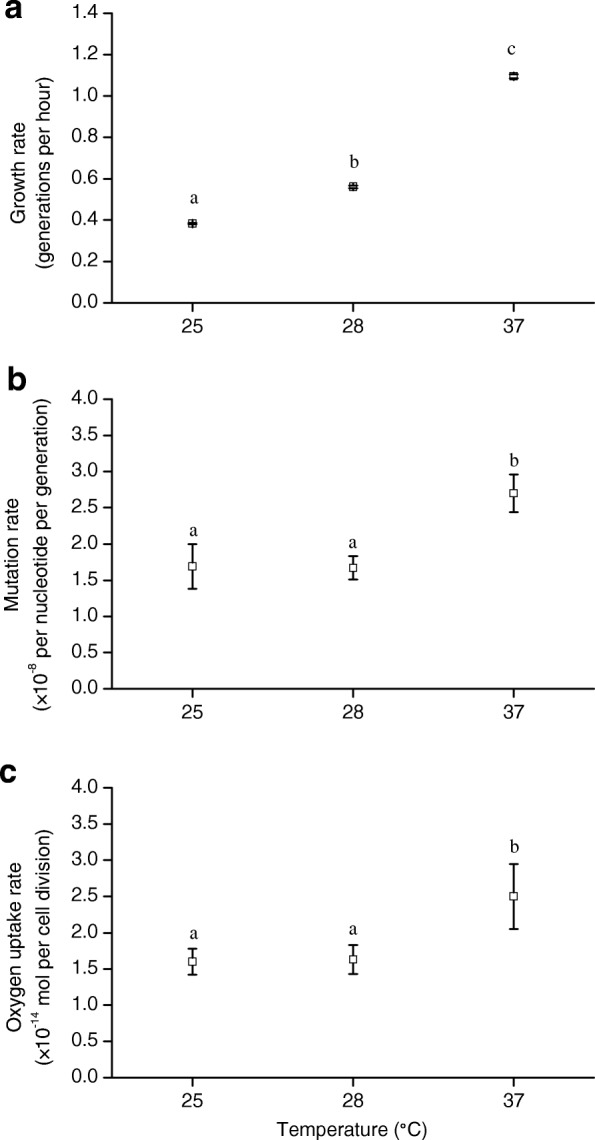
Bacterial growth rate, mutation rate and metabolic rate at three temperatures. **a** Growth rate (sample size *N* = 20 colonies for each temperature). **b** Mutation rate (*N* = 20 lines for each temperature). **c** Metabolic rate (oxygen uptake rate; *N* = 14, 11, and 12 test agar plates at 25, 28 and 37 °C, respectively). In each panel, data show mean ± 95% CL, where the 95% CLs were given by multiplying SEMs by the critical value of the *t* distribution; and data points annotated with a same letter had no significant difference (*P*_adj_ > 0.05; based on *t* tests, with *P* values from multiple comparisons between temperatures for each data set corrected using the Benjamini-Hochberg procedure)

We carried out a mutation accumulation experiment with *E. coli* B REL606 *mutS*, a mutator strain with a disrupted allele of *mutS* that is involved in an unbiased DNA repair pathway. The experiment was performed at three temperatures, 25, 28 and 37 °C. All the three temperatures are within the normal temperature range, and non-stressful, for our study species; 37 °C is a within-host temperature, and 25 and 28 °C are common temperature conditions faced with this species in natural soil and water habitats, and none of the three temperatures is near the lower (19 °C) or upper temperature limits (42 °C) of our study strain [38, 39]. The three temperatures differed significantly in affecting bacterial growth rate (number of generations per hour; Fig. 1a; 25 versus 28 °C, *t* = − 48.36, df = 38, *P*_adj_ < 10^− 10^; 25 versus 37 °C, *t* = − 138.64, df = 38, *P*_adj_ < 10^− 10^; 28 versus 37 °C, *t* = − 89.30, df = 38, *P*_adj_ < 10^− 10^). Growth rates of the bacterium at 25 and 28 °C were 0.65- and 0.49-fold lower than that at 37 °C, respectively.

### Mutation rate

The rate of mutation (number of mutations per nucleotide per generation, including both spontaneous base pair substitutions and small indels), showed no significant difference between 25 and 28 °C, but was higher at 37 °C (Table S1; Fig. 1b; 25 versus 28 °C, *t* = 0.08, df = 29, *P*_adj_ = 0.469; 25 versus 37 °C, *t* = − 5.26, df = 32, *P*_adj_ = 7 × 10^− 6^; 28 versus 37 °C, *t* = − 7.12, df = 33, *P*_adj_ = 6 × 10^− 8^). Specifically, MA lines from 25 and 28 °C showed mutation rates 0.37- and 0.38-fold lower than that from 37 °C, respectively.

### Spectrum of base pair substitutions

**Fig. 2:**
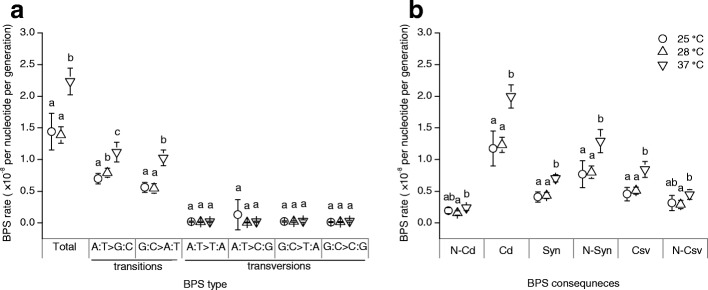
Mutation rates of BPSs at three temperatures. **a** Rates of total, and the six types of, BPSs. **b** Rates of BPSs categorized based on consequences. Cd, coding; Csv, conservative; N-Cd, noncoding; N-Csv, non-conservative; N-Syn, nonsynonymous; Syn, synonymous. In each panel, data show mean ± 95% CL, where the 95% CLs were given by multiplying SEMs by the critical value of the *t* distribution. Within each category of BPSs, data points annotated with a same letter had no significant difference (*P*_adj_ > 0.05; based on *t* tests, with *P* values from multiple comparisons between temperatures for each data set corrected using the Benjamini-Hochberg procedure)

The base pair substitutions (BPS) spectrum was biased to transitions, as reported before [40, 41]; and this bias was observed at all temperatures. Transitions account for 87% of all the BPSs at 25 °C, 96% at 28 °C and 96% at 37 °C. Rates of total BPSs, total transitions, A:T > G:C transitions, G:C > A:T transitions were all higher at 37 °C than 25 and 28 °C, while the latter two temperatures only differed in the incidence of A:T > G:C transitions.. Meanwhile the incidences of transversions did not show obvious differences among temperatures (Fig. 2a; Additional file 1: Table S1).

More detailed analysis of BPS spectrum was presented in Supporting Information (Additional file 1: Text S1; Table S1-S5), and two findings are highlighted here. First, MA lines showed coding/noncoding BPS ratios higher than expected at 37 °C, but not 25 or 28 °C (Fig. 2b, Additional file 1: Table S2). This suggests a temperature dependence in the relative susceptibility to mutation in coding versus noncoding DNA in the study organism. Second, the ratio of nonsynonymous to synonymous mutations in coding DNA did not differ significantly from the expected value (Fig. 2b, Additional file 1: Table S2), suggesting a negligible effect of selection during the MA experiment, consistent with previous studies [36, 37, 42].

### Spectrum of small indels

**Fig. 3:**
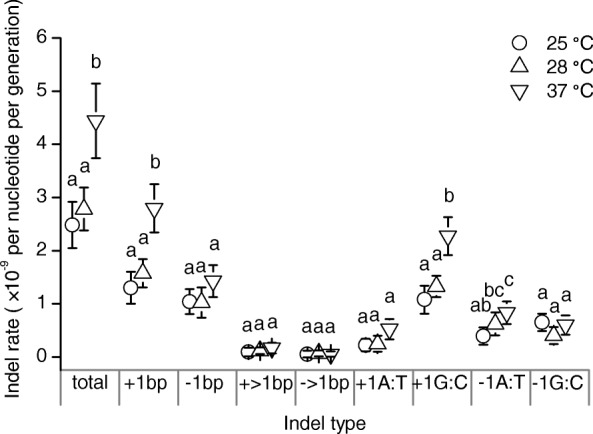
Rates of indels at three temperatures. Bp, base pair. Data show mean ± 95% CL, where the 95% CLs were given by multiplying SEMs by the critical value of the *t* distribution. Within each category of indels, data points annotated with a same letter had no significant difference (*P*_adj_ > 0.05; based on *t* tests, with *P* values from multiple comparisons between temperatures for each data set corrected using the Benjamini-Hochberg procedure)

Small indels rate was around 1/5th of BPSs rate in the present study. Base pair gains were more frequent than losses at all the three temperatures. Significant differences between temperatures (usually higher values at 37 °C than the other two temperatures) were observed for the following types of indels: total indels, 1 bp insertion, + 1 G:C, − 1 A:T, − 1 G:C (Fig. 3; Additional file 1: Table S6). Sequence repeats are hotspots for indels as reported before [37, 43], and the indels rate in runs was significantly higher at 37 °C in this study (Additional file 1: Table S6).

### Metabolic rate, and the similarity with mutation rate in temperature response

Metabolic rate was measured as oxygen uptake rate (mol O_2_ per cell division), with higher values suggesting more energy consumption per generation of growth. A significant difference in metabolic rate was observed between 37 °C and the other two temperatures, but not between 25 and 28 °C (Fig. 1c; 25 versus 28 °C, *t* = − 0.58, df = 16, *P*_adj_ = 0.568; 25 versus 37 °C, *t* = − 3.22, df = 14, *P*_adj_ = 0.018; 28 versus 37 °C, *t* = − 2.50, df = 19, *P*_adj_ = 0.033). Compared with 37 °C, the 25 and 28 °C environments had 0.36- and 0.34-fold lower metabolic rates, respectively.

The rate of mutation (including both BPS and small indels) showed a temperature dependence highly similar to that of metabolic rate (Fig. 1b-c). The ratio of mutation rate to metabolic rate, i.e., mutation rate expressed as number of mutations per nucleotide per mol O_2_ consumption, was highly similar across the three temperatures (1.06, 1.02 and 1.08 × 10^6^ at 25, 28 and 37 °C, respectively).

## Discussion

The present study provided a reliable and accurate estimate for the temperature effects on spontaneous mutation in an *E. coli* strain. In particular, the approach of MA combined with WGS can give a complete and unbiased description about spontaneous mutations, and therefore we may overcome some major methodological issues in previous studies that relied on reporter genes for mutation rate measurement [31, 37, 42, 44]. Moreover, population passage at the same growth phase across temperatures (similar sizes of colonies on agar plates) ruled out the possibility that temperature effects on mutation is obscured by variation in nutrient limitation, which itself may have mutagenic effects [28, 45–47]. We used a mutator strain (*E. coli* B REL606 *mutS*), allowing a relatively short MA experiment to efficiently reveal the temperature effects on mutation rate and mutational spectrum. Our study strain had a disrupted allele of *mutS* that is involved in an unbiased DNA repair pathway (with other mismatch repair functions being intact), and was suggested to show a mutation rate ~ 30 times higher than the wild-type strain [48]. Our results confirmed a positive temperature dependence of mutation rate, within a normal (non-stressful) temperature range for the study organism, while earlier studies usually reported mutagenic effects of extremely high temperatures. We also found highly similar temperature responses between mutation rate and metabolic rate. There might exist a causal link between mutation and metabolism, if oxidative DNA damage functions as the major source of mutation and its occurrence is determined by metabolic rate under aerobic conditions [3, 37, 49–51]. Alternatively, this may only reflect a correlation, where the two sources of spontaneous mutation, oxidative DNA damage and replication errors, both show temperature responses similar with metabolic rate (e.g. [52, 53]). Therefore, the possibility that metabolic rate predicts mutation rate deserves further research; for example, experiments that directly manipulate metabolic activity or metabolic by-products without affecting other major determinants of spontaneous mutations would better solve this question.

Temperature may also alter the biases in mutational spectrum, and thus the consequences of mutations. Transitions accounted for the majority of BPSs observed in our MA lines; and the incidence of both types of transitions was higher at 37 °C relative to 25 and 28 °C. Meanwhile, A:T > G:C (but not G:C > A:T) transitions occurred more frequently at 28 relative to 25 °C. The occurrence of transversions was not affected by temperature (Fig. 2a; Additional file 1: Table S1). This is inconsistent with an earlier suggestion that oxidation of guanine to 8-oxoG (leading to A:T > C:G transversion) is a major source of oxidative DNA damage in *E. coli* [37, 42]. However, the results could be explained by the temperature sensitivity of cytosine deamination, as deamination of cytosine to uracil is a cause of G:C > A:T transition [54]. The latter explanation is also consistent with a finding that cytosine is the primary site affected by oxidative stress-induced mutagenesis [51], considering that the higher temperatures cause greater oxidative stress. The increasing A:T > G:C transition rate with temperature implies a possibility that the production or activity of mutagenic agents inducing this type of transition (including O^6^-methylguanine and 5-bromouracil [55, 56]) was sensitive to temperature. Meanwhile, we cannot rule out the possibility that temperature has regulated the efficiency of the mismatch repair functions and, by which, contributed to these changes in mutational spectrum.

The MA lines from 37 °C showed a bias of BPSs toward coding, versus noncoding, DNA, whereas this pattern was not found for the two lower temperatures. This does not mean that the role of selection during the MA experiment has differed among temperatures. Indeed, analysis of the ratio of nonsynonymous to synonymous mutations in coding DNA suggested a negligible role of selection at all the three experimental temperatures. The bias in coding versus noncoding DNA mutation therefore does indicate a significant role of temperature for shaping mutational heterogeneity across different regions of the genome in the study strain, which might be caused by the temperature dependence of the function and target of DNA repair machinery. We did not investigate the differences in mutation bias between highly and less highly expressed genes, as gene expression level is a quantitative trait and may vary with temperature while data available from public databases are generally obtained from assays at only specific temperatures (particularly 37 °C). Nonetheless, it is interesting to ask how temperature, or other physical environmental factors, modulates the effect of physiological processes in determining mutation heterogeneity (e.g. a bias in DNA mismatch repair functions toward coding DNA due to stronger selection to reduce mutations in coding than noncoding regions; [57, 58]).

The finding that temperature altered the biases in coding versus noncoding DNA has crucial implications for our understanding of the temperature effect on the speed of adaptive evolution. Three major mechanisms were proposed for explaining the effect of higher temperatures for accelerating evolutionary processes: shorter generation times, higher mutation rate and stronger natural selection [8, 9, 16]. While the former two mechanisms received much attention, the ‘selection strength’ hypothesis has been largely overlooked. Theoretical possibilities proposed several decades ago included that warmer environments allow a wider range of mutations to confer fitness effects (that is, fitness consequence of a given mutation is conditional on environmental temperature), and that temperature affects population demography or species interactions and thus indirectly alters the strength of selection [8, 9, 59]. Our results suggest another possibility: higher temperatures increase the supply of mutations with phenotypic effects.

Appropriate caution should be taken in drawing any broad conclusions from our findings. First, we worked with a bacterial strain with reduced mismatch repair function, thus our suggestion that increasing temperatures may generally elevate mutation rate is to some extent tentative (as it is unknown how the *mutS* function missing in our study strain is regulated by temperature). Second, the hypothesized link between metabolic activity and mutation rate may not apply equally well in all taxa. Particularly, DNA is in the form of compact chromosomes separated from the rest of the cell and thus is protected from oxidative damage to a certain extent in eukaryotes, but not in prokaryotes. More research is obviously needed to address the generality of the temperature effects on spontaneous mutation and the metabolic rate-mutation rate relationship [53].

## Conclusions

Our study showed that temperature could drive both mutation rate and the types of mutation supply, contributing to a more comprehensive understanding of how temperature determines the chance of natural selection and thus the speed of evolution. Our results also lend support to the metabolic theory of ecology for linking metabolic rate and molecular evolution rate. Knowledge of the temperature response of spontaneous mutation has crucial implications not only for understanding the evolutionary causes of biodiversity patterns [10, 12], but also for resolving challenges related to contemporary evolution such as predicting the emergence of new infectious diseases [60, 61].

## Methods

### The mutation accumulation experiment

The bacterial strain used in this study was *E. coli* B REL606 *mutS*, a mutator derivative of the wild-type strain. It was constructed by P1 transduction of a disrupted allele of *mutS*, *mutS*::Tn5, into REL606 [62]. The *mutS* protein is a major component of the mismatch repair system in *E. coli*, involved in recognizing and binding to mispaired nucleotides (and thus initiating the repair processes). Thus the *mutS* is involved in an unbiased DNA repair pathway [63], unlike many other mismatch repair functions including *mutT*, *mutY* and *mutM*, which target specific types of mutations [37]. The other mismatch repair functions are intact in this strain. Therefore, it is likely that disruption of the *mutS* function may increase the mutation rate but not result in large differences in mutational spectrum. To work with this mismatch repair deficient bacterial strain would allow a relatively short MA experiment to efficiently detect spontaneous mutations. Lysogeny broth (LB) medium was used for bacterial culture [48]. To make frozen stocks, bacteria were grown in LB broth, and mixed with glycerol (1:1 in volume), and then stored at − 80 °C.

A single colony of the ancestral strain was inoculated to LB medium and grown overnight at 37 °C, dilutions of which were spread onto LB agar plates and grown at three temperatures, 25, 28 and 37 °C. Then 60 mutation accumulation (MA) lines were established, 20 replicates at each of the three temperatures. For each MA line, a single colony was streaked onto a new agar plate for every round of population growth, which involved 72 h of incubation at 25 °C, 48 h at 28 °C, or 24 h at 37 °C (this incubation regime led to similar colony size, and thus similar numbers of generations per passage, across temperatures). After each round of colony growth, a single, well-isolated, colony was randomly picked up and used to streak a new agar plate. To ensure random choosing of the colony for passage for each MA line, we picked up a well-isolated colony closest to a mark drawn on the edge of the plate. All the 60 MA lines went through 30 bottlenecks. Bacterial samples were frozen stored every 10 bottlenecks.

The number of generations undergone by each MA line per passage was determined based on the number of cells in the colonies (log_2_ of cell numbers per colony, usually 27 generations). This involved making a suspension of the colony with NaCl solution (1%) and spreading dilutions onto LB agar plates, and counting the colony forming units after 24 h incubation at 37 °C. Measurement of colony sizes was carried out at bottleneck 1, 11, and 21 for each MA lines, which were used for estimating the number of generations elapsed during bottleneck 1–10, 11–20, and 21–30, respectively. Growth rate of the ancestral strain at each temperature was also estimated by measuring colony sizes [38].

### Whole-genome sequencing and analysis

Genomic DNA was extracted for the ancestral strain and the 60 MA lines at bottleneck 30. The TIANamp Bacteria DNA Kit (TIANGEN) was used for extracting and purifying DNA from overnight bacterial cultures in LB broth inoculated with frozen stocks. DNA concentration and purity were assessed with a Qubit3.0 Fluorometer (Thermo Fisher Scientific Inc., Waltham, MA, USA) and Nanophotometer P330 (IMPLEN Inc., Westlake Village, CA 91362, USA).

Whole-genome sequencing of the ancestral *E. coli* B REL606 *mutS* was performed using PacBio third-generation sequencing at Novogene. The sequenced library contained 103,174 reads, with a mean length of 14,638 bp and N50 length of 20,782 bp. Reads were assembled by SMRT portal, yielding a complete circular genome of 4,643,117 bp with GC content of 50.77%.

For the MA lines, library construction and resequencing were conducted at Novogene, using the PE150 platform (Illumina HiSeq X ten). For quality-control purposes, reads with any one of the following characteristics were discarded: (i) ≥ 10% Ns; (ii) ≥ 50% low-quality (Q ≤ 5) bases; (iii) adapter contamination. The following trimming strategies were performed to cut the low-quality reads: (i) minimal quality of remained bases ≥20; (ii) length of reads after trimming ≥65. After such filtering, an average depth of 163× of each line was retained. Note that we did not use the published genome of *E. coli* B REL606 [64] as a reference here, considering the possibility that our ancestral *mutS* strain may have had substantial sequence difference from the wild-type strain.

Trimmed reads were mapped to the ancestral genome with Burrows-Wheeler Aligner BWA-MEM algorithm, version 0.7.8 [65]. BPSs and short indels (≤ 4 bp) were called with SAMTOOLS, version 0.1.19 [66]. To minimize the impact of sequencing errors, we excluded sites for which read depth was less than 20×. The sequences and mutations reported in this study have been submitted to the NCBI Sequence Read Archive (BioProject Accession number PRJNA383601).

Protein-coding genes were predicted by GeneMarkS [67], and the predicted genes were run searches against the Swiss-Prot database by BLAST to annotate their functions. Variants were annotated based on the predicted genes. BPSs in coding sequences were determined to be synonymous or nonsynonymous based on the Bacterial, Archaeal and Plant Plastid Code at NCBI (https://www.ncbi.nlm.nih.gov/Taxonomy/Utils/wprintgc.cgi?mode=t#SG11). Nonsynonymous BPSs were designated conservative or non-conservative based on the BLOSUM62 score matrix, with a value ≥0 considered conservative.

### Metabolic rate measurement

Oxygen uptake rate (OUR) was measured as an estimate of metabolic rate of the ancestral strain [68, 69], as the energy yield of aerobic oxidation of a given substrate is a constant [70]. Airtight containers were used to measure the OUR of bacterial cells on agar plates. Each container has inner space that was just larger than a 9 cm Petri-Dish, and a lid with a rubber-sealed port (Additional file 1: Figure S1). Cultures of the ancestral strain were grown overnight in LB broth at 37 °C, 1 ml of which was transferred to 10 ml of fresh LB broth and grown for 24 h at each of the following temperatures, 25, 28, and 37 °C. Dilutions of the cultures were spread onto LB agar plates, which were then placed in the airtight containers and grown for certain periods of time (72 h at 25 °C, 48 h at 28 °C, or 24 h at 37 °C). Oxygen concentrations in the container were measured using Head Space Analyser EasyCheck Two (ADEV Inc., Cesano Maderno, Italy) before and after incubation, with air samples collected through needles puncturing the rubber seal of the containers. Volume of gas in each container filled with an agar plate was estimated by injecting volume-known water into the system. The number of cell divisions for each assay were calculated based on the number of colonies and the number of cells per colony (for a colony of *N* cells, the number of cell divisions is *N* - 1). Data from assays where more than 4 pairs of colonies were not well-isolated were discarded. The OUR was expressed as the amount of oxygen uptake per cell division.

### Data analysis

To obtain the expected parameters for mutational spectra, we carried out Monte Carlo simulations for a random distribution of BPSs. With the number of mutations being fixed at the observed numbers and the ratio of six types of BPSs set as the observed values at each temperature, 1000 trials were simulated [36]. Custom scripts used to examine the number of variants in different features, as well as those to generate the random BPSs data with Monte Carlo Simulation, are available online (https://github.com/zhangbw3187/spontaneousmutation). Standard statistical analyses, including one-tailed *t* test and Chi-squared test, were used for data analysis. The Benjamini-Hochberg procedure was used for correcting *p* values obtained from multiple comparisons [71].

## Additional file

Additional file 1: Additional results and discussion. (DOCX 326 kb)

## Abbreviations

BPS: Base pair substitution
ESH: Evolutionary speed hypothesis
MA: Mutation accumulation
OUR: Oxygen uptake rate
WGS: Whole-genome sequencing
