# Supplementary material for: Temperature responses of mutation rate and mutational spectrum in an Escherichia coli strain and the correlation with metabolic rate
Source: BMC Evol Biol. 2018 Aug 29;18:126. doi: 10.1186/s12862-018-1252-8 (PMC6116381; doi:10.1186/s12862-018-1252-8)
Supplement: Supplementary file 1 — Additional results and discussion. (DOCX 326 kb) [file 12862_2018_1252_MOESM1_ESM.docx]

**Additional Information for:**

**Temperature responses of mutation rate and mutational spectrum in an *Escherichia coli* strain and the correlation with metabolic rate**

**Additional Text S1** Additional results and discussion

In our MA lines, the A:T > G:C transition occurred more frequently than G:C > A:T (Fig. 2a, Table S1), the same as in several mismatch repair deficient strain reported before [1,2], but contrary to earlier observations in non-KO (knocked-out) MA lines that showed a bias in transitions toward G:C > A:T (strong hydrogen bond to weak hydrogen bond) for many eukaryotes and prokaryotes [3].

Biases caused by local sequence context were observed in mutation frequencies for MA line from all the three experimental temperatures. Particularly, transitions at A:T sites in the sequence 5’**A**pC3’ or the equivalent 3’**T**pG5’ were 2.70-fold more frequent than expected from the frequency of this dimer in the genome (note: the mutated bases are underlined and in bold; the “p” means the phosphate linking the two nucleotide). This implies a prominent role of neighboring C or G of A:T sites in affecting A:T > G:C transitions, consistent with earlier studies [1,2]. By contrast, the transitions at A:T sites in the context 5’**A**pA3’/3’**T**pT5’ were less frequent by around 0.28-fold relative to the expected values, while those in the context 5’**A**pT3’/3’**T**pA5’ were about 0.45-fold less frequent. The transitions at G:C sites in the context 5’Ap**G**3’/3’Tp**C**5’were also nearly 0.40-fold less frequent than expected. In the context of 5’Cp**G**3’/3’Gp**C**5’, G:C > A:T transitions occurred more frequently than expected at 28 °C and 37 °C, but not 25 °C; in the context of 5’**G**pC3’/3’**C**pG5’, the G:C > A:T transitions were more frequent than expected only at 37 °C; and in the context of 5’**G**pA3’/3’**C**pT5’, the G:C > A:T transitions were less frequent than expected only at 28 °C (Table S3).

BPSs showed a strong DNA-strand bias at all the three temperatures. For example, G:C > A:T transitions were twice as likely to occur with C templating the lagging strand and G templating the leading strand during replication than *vice versa*; A:T > C:G transitions were nearly twice as likely to occur with A templating the lagging strand and T templating the leading strand (Table S4).

Differences were also observed between transcribed versus nontranscribed strands in transition rate. The A > G transition rate was biased to the nontranscribed strand at 37 °C, while G > A transition rate biased to the transcribed strand at 25 °C and 37 °C. The biases also exist for total BPSs occurred at A and G sites at 25 °C and 37 °C. The total SNPs at A site biased to nontranscribed strand at 28 °C (Table S5).

In a previous study with an *E. coli* K12 strain, G**A**TC sequences were found to be hotspots for A:T transversions [2], possibly because 6meA is prone to depurination, producing tranversions [4]. The same hotspots were also found in the strain in our experiment, only 0.8% of the total A:T base pairs occur in the G**A**TC sites, but accounted for 1.8% (2/114) of the A:T transversions at 25 °C, 25% (6/24) at 28 °C, and 11% (3/27) at 37 °C. These proportions are significantly higher than expected at 28 °C and 37 °C, although non-significantly at 25 °C (*χ*^2^ = 1.1, *P* = 0.29; *χ*^2^ = 134.6, *P* ~ 0; *χ*^2^ = 30.8, *P* = 2×10^-8^, for 25, 28 and 37 °C, respectively).

We did not find the internal Cs mutational hotspots for G:C to A:T in the sequences C**C**WGG as reported before [2], possibly because our *E. coli* B derivative strain lacks the methylation system responsible for the transition hotspots [5,6].

**Additional Fig. S1** Picture of a container for measurement of Oxygen Uptake Rate (OUR).


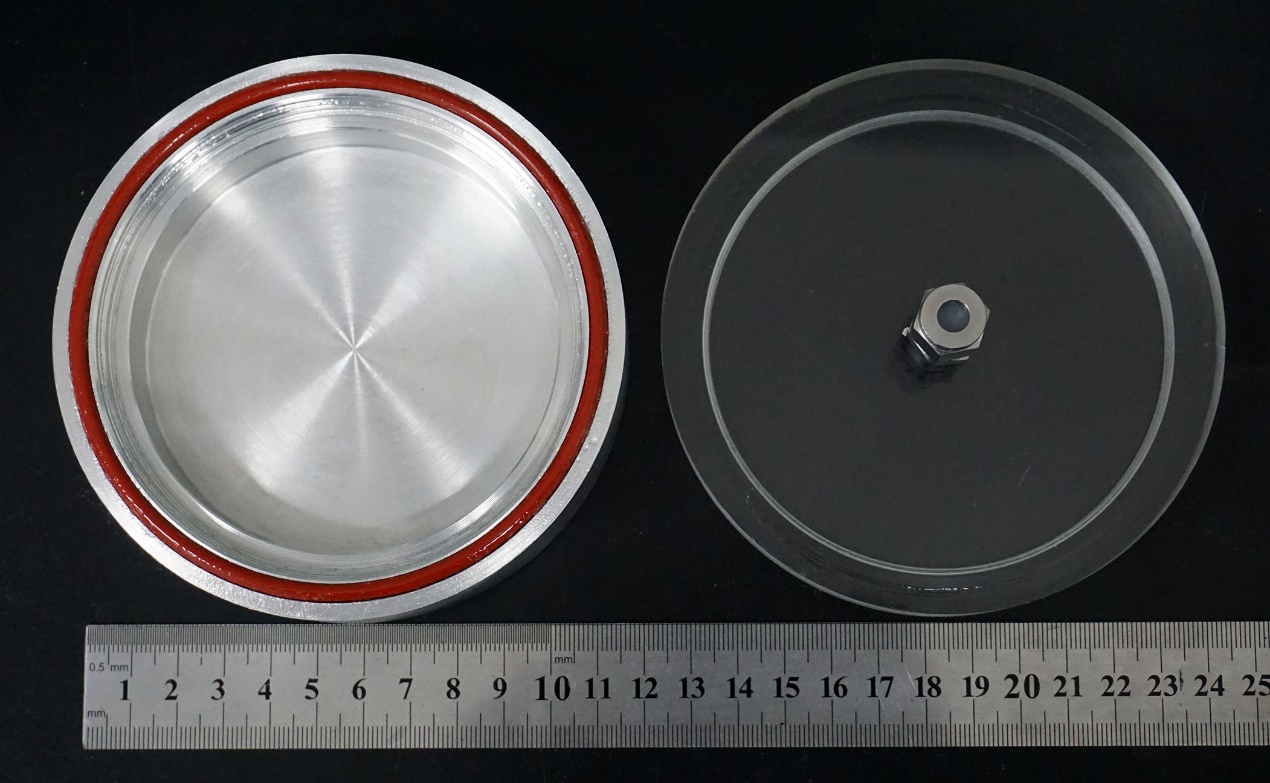


**Additional Table S1** BPS spectra at three temperatures

| mutational changes | 25℃ |  | 28℃ |  | 37℃ |
| --- | --- | --- | --- | --- | --- |
| Type of substitution |  |  |  |  |  |
| Total | 1 108 ^a^ |  | 1 041 ^a^ |  | 1 636 ^b^ |
| Transitions | 969 ^a^ |  | 1 002 ^a^ |  | 1 569 ^b^ |
| A:T > G:C | 537 ^a^ |  | 594 ^b^ |  | 818 ^c^ |
| G:C > A:T | 432 ^a^ |  | 408 ^a^ |  | 751 ^b^ |
| Transversions | 139 ^a^ |  | 39 ^a^ |  | 67 ^a^ |
| A:T > T:A | 15 ^a^ |  | 11 ^a^ |  | 14 ^a^ |
| A:T > C:G | 99 ^a^ |  | 4 ^a^ |  | 13 ^a^ |
| G:C > T:A | 17 ^a^ |  | 15 ^a^ |  | 21 ^a^ |
| G:C > C:G | 8 ^a^ |  | 9 ^a^ |  | 19 ^a^ |
| A:T sites | 651 ^a^ |  | 609 ^a^ |  | 845 ^b^ |
| G:C sites | 457 ^a^ |  | 432 ^a^ |  | 791 ^b^ |
| Consequences of substitutions | | | | | |
| Position |  |  |  |  |  |
| N-Cd | 154 ^ab^ |  | 117 ^a^ |  | 175 ^b^ |
| Cd | 954 ^a^ |  | 924 ^a^ |  | 1461 ^b^ |
| Within coding sequences | | | | | |
| Syn | 331 ^a^ |  | 324 ^a^ |  | 515 ^b^ |
| N-Syn | 623 ^a^ |  | 600 ^a^ |  | 946 ^b^ |
| Amino acid changes | | | | | |
| Csv | 699 ^a^ |  | 706 ^a^ |  | 1133 ^b^ |
| N-Csv | 255 ^ab^ |  | 218 ^a^ |  | 328 ^b^ |

Cd, coding; Csv, conservative; N-Cd, noncoding; N-Csv, non-conservative; N-Syn, nonsynonymous; Syn, synonymous. Within each row, numbers annotated with different letters had significant difference (*P*_adj_ < 0.05; based on *t* tests, with *P* values from multiple comparisons between temperatures for each data set corrected using the Benjamini-Hochberg procedure).

**Additional Table S2** Observed BPSs and amino acid changes at three temperatures compared with expected.

| Mutational change | 25°C | | |  | 28°C | | |  | 37°C | | | |
| --- | --- | --- | --- | --- | --- | --- | --- | --- | --- | --- | --- | --- |
|  | Obs | Exp | *P* |  | Obs | Exp | *P* |  | Obs | Exp | *P* |  |
| Using fractions of all BPSs | | | | | | | | | | | | |
| G:Cs | 457 | 563 |  |  | 432 | 529 |  |  | 791 | 831 |  |  |
| A:Ts | 651 | 545 |  |  | 609 | 512 |  |  | 845 | 805 |  |  |
| G:C/A:Ts | 0.70 | 1.03 | 1×10^-10 ***^ |  | 0.71 | 1.03 | 2×10^-9^ ^***^ |  | 0.94 | 1.03 | 0.048 ^*^ |  |
|  |  |  |  |  |  |  |  |  |  |  |  |  |
| G:C Ts | 432 | 492 |  |  | 408 | 509 |  |  | 751 | 797 |  |  |
| A:T Ts | 537 | 477 |  |  | 594 | 493 |  |  | 818 | 772 |  |  |
| G:C Ts/A:T Ts | 0.80 | 1.03 | ~0 ^***^ |  | 0.69 | 1.03 | 2×10^-10^ ^***^ |  | 0.92 | 1.03 | 0.02 ^*^ |  |
|  |  |  |  |  |  |  |  |  |  |  |  |  |
| G:C Tr | 25 | 71 |  |  | 15 | 19 |  |  | 40 | 34 |  |  |
| A:T Tr | 114 | 68 |  |  | 24 | 20 |  |  | 27 | 33 |  |  |
| G:cTr/A:T Tr | 0.22 | 1.04 | ~0 ^***^ |  | 0.63 | 0.95 | 0.2 |  | 1.48 | 1.03 | 0.14 |  |
|  |  |  |  |  |  |  |  |  |  |  |  |  |
| Cd | 954 | 970 |  |  | 924 | 912 |  |  | 1 461 | 1 433 |  |  |
| N-Cd | 154 | 138 |  |  | 117 | 129 |  |  | 175 | 203 |  |  |
| Cd/N-Cd | 6.20 | 7.03 | 0.15 |  | 7.90 | 7.07 | 0.26 |  | 8.35 | 7.06 | 0.04 ^*^ |  |
| Using fractions of BPSs in coding DNA | | |  |  |  |  |  |  |  |  |  |  |
| N-Syn | 623 | 1 117 |  |  | 600 | 707 |  |  | 946 | 730 |  |  |
| Syn | 331 | 344 |  |  | 324 | 217 |  |  | 515 | 224 |  |  |
| N-Syn/Syn | 1.88 | 3.25 | ~0 ^***^ |  | 1.852 | 3.25 | ~0 ^***^ |  | 1.837 | 3.25 | ~0 ^***^ |  |
| Using fractions of N-Syn changes | |  |  |  |  |  |  |  |  |  |  |  |
| N-Csv | 255 | 294 |  |  | 218 | 283 |  |  | 328 | 447 |  |  |
| Csv | 368 | 329 |  |  | 382 | 317 |  |  | 618 | 499 |  |  |
| N-Csv/Csv | 0.69 | 0.89 | 2×10^-3^ ^**^ |  | 0.57 | 0.89 | 1×10^-7^ ^***^ |  | 0.53 | 0.90 | ~0 ^***^ |  |
| Using simulations |  |  |  |  |  |  |  |  |  |  |  |  |
| Cd | 954 | 965 |  |  | 924 | 907 |  |  | 1 461 | 1430 |  |  |
| N-Cd | 154 | 143 |  |  | 117 | 134 |  |  | 175 | 206 |  |  |
| Cd/N-Cd | 6.20 | 6.75 | 0.32 |  | 7.897 | 6.77 | 0.12 |  | 8.349 | 6.94 | 0.02 ^*^ |  |
|  |  |  |  |  |  |  |  |  |  |  |  |  |
| N-Syn | 623 | 649 |  |  | 600 | 614 |  |  | 946 | 966 |  |  |
| Syn | 331 | 305 |  |  | 324 | 310 |  |  | 515 | 495 |  |  |
| N-Syn/Syn | 1.882 | 2.13 | 0.07 |  | 1.852 | 1.98 | 0.33 |  | 1.837 | 1.95 | 0.27 |  |
|  |  |  |  |  |  |  |  |  |  |  |  |  |
| N-Csv | 255 | 285 |  |  | 218 | 263 |  |  | 328 | 638 |  |  |
| Csv | 368 | 338 |  |  | 382 | 337 |  |  | 618 | 823 |  |  |
| N-Csv/Csv | 0.69 | 0.84 | 0.02 ^*^ |  | 0.57 | 0.78 | 2×10^-4^ ^***^ |  | 0.53 | 0.78 | 2×10^-8^ ^***^ |  |

For BPSs comparisons using fractions, the expected values were obtained from the fraction of possible BPS or amino acid changes of each type based on the genome. For BPSs comparisons using simulations, the expected values were calculated using the means of 1,000 Monte Carlo simulations using the spectrum of BPSs at each temperature. Ts, transitions; Tv, transversions; Cd, coding DNA; N-Cd, noncoding DNA; N-Syn, nonsynonymous base-pair change; Syn, synonymous base-pair change; N-Csv, non-conservative amino acid change; Csv, conservative amino acid change; Obs, observed number of mutations; Exp, expected value of BPSs; *P*, probability calculated for the Chi-square test for the two observed values versus the two expected values. *P*~0 is used to indicate probabilities < 10^-10^, and asterisks indicate significant differences as follows: ^***^*P* ≤ 0.001; ^**^*P* ≤ 0.01; ^*^*P* ≤ 0.05.

**Additional Table S3** Local sequence biases of transition mutations at three temperatures.

|  | 25°C | | | |  | 28°C | | | |  | 37°C | | | |
| --- | --- | --- | --- | --- | --- | --- | --- | --- | --- | --- | --- | --- | --- | --- |
| 5`NMN3`/3`NMN5` | Obs | Exp | Obs/Exp | *P* |  | Obs | Exp | Obs/Exp | *P* |  | Obs | Exp | Obs/Exp | *P* |
| G**G**N/C**C**N | 112 | 99 | 1.13 | 0.37 |  | 116 | 94 | 1.23 | 0.13 |  | 221 | 173 | 1.28 | 0.02 ^*^ |
| C**G**N/G**C**N | 168 | 127 | 1.32 | 0.02 ^*^ |  | 178 | 120 | 1.48 | 8×10^-4^ ^***^ |  | 299 | 221 | 1.35 | 6×10^-4^ ^***^ |
| A**G**N/T**C**N | 37 | 87 | 0.43 | 7×10^-6^ ^***^ |  | 33 | 82 | 0.40 | 5×10^-6^ ^***^ |  | 56 | 151 | 0.37 | ~0 ^***^ |
| T**G**N/ACN | 115 | 119 | 0.97 | 0.79 |  | 81 | 112 | 0.72 | 0.03 ^*^ |  | 175 | 206 | 0.85 | 0.11 |
|  | 432 | 432 |  | 2×10^-9^ ^***^ |  | 408 | 408 |  | ~0 ^***^ |  | 751 | 751 |  | ~0 ^***^ |
|  |  |  |  |  |  |  |  |  |  |  |  |  |  |  |
| N**G**G/N**C**C | 105 | 99 | 1.06 | 0.67 |  | 113 | 94 | 1.20 | 0.19 |  | 163 | 173 | 0.94 | 0.59 |
| N**G**C/N**C**G | 181 | 141 | 1.28 | 0.03 ^*^ |  | 178 | 133 | 1.34 | 0.01 ^**^ |  | 331 | 245 | 1.35 | 3×10^-4^ ^***^ |
| N**G**A/N**C**T | 69 | 98 | 0.70 | 0.02 ^*^ |  | 51 | 93 | 0.55 | 5×10^-4^ ^***^ |  | 131 | 170 | 0.77 | 0.02 ^*^ |
| N**G**T/N**C**A | 77 | 94 | 0.82 | 0.19 |  | 66 | 89 | 0.74 | 0.06 |  | 126 | 163 | 0.77 | 0.03 ^*^ |
|  | 432 | 432 |  | 3×10^-5^ ^***^ |  | 408 | 408 |  | 1×10^-9^ ^***^ |  | 751 | 751 |  | 2×10^-10^ ^***^ |
|  |  |  |  |  |  |  |  |  |  |  |  |  |  |  |
| G**A**N/C**T**N | 152 | 126 | 1.21 | 0.12 |  | 160 | 139 | 1.15 | 0.22 |  | 218 | 191 | 1.14 | 0.18 |
| C**A**N/G**T**N | 115 | 152 | 0.76 | 0.02 ^*^ |  | 128 | 168 | 0.76 | 0.02 ^*^ |  | 206 | 232 | 0.89 | 0.21 |
| A**A**N/T**T**N | 156 | 159 | 0.98 | 0.87 |  | 186 | 176 | 1.06 | 0.60 |  | 195 | 243 | 0.80 | 0.02 ^*^ |
| T**A**N/A**T**N | 114 | 100 | 1.14 | 0.34 |  | 120 | 110 | 1.09 | 0.51 |  | 199 | 152 | 1.31 | 0.01 ^**^ |
|  | 537 | 537 |  | 9×10^-4^ ^***^ |  | 594 | 594 |  | 0.003 |  | 818 | 818 |  | 1×10^-6^ ^***^ |
|  |  |  |  |  |  |  |  |  |  |  |  |  |  |  |
| N**A**G/N**T**C | 110 | 111 | 0.99 | 0.95 |  | 115 | 123 | 0.93 | 0.60 |  | 113 | 170 | 0.66 | 7×10^-4^ ^***^ |
| N**A**C/N**T**G | 327 | 120 | 2.73 | ~0 ^***^ |  | 360 | 133 | 2.71 | ~0 ^***^ |  | 531 | 183 | 2.90 | ~0 ^***^ |
| N**A**A/N**T**T | 42 | 159 | 0.26 | ~0 ^***^ |  | 51 | 176 | 0.29 | ~0 ^***^ |  | 67 | 243 | 0.28 | ~0 ^***^ |
| N**A**T/N**T**A | 58 | 146 | 0.40 | 7×10^-10^ ^***^ |  | 68 | 161 | 0.42 | 8×10^-10^ ^***^ |  | 107 | 222 | 0.48 | 2×10^-10^ ^***^ |
|  | 537 | 537 |  | 5×10^-7^ ^***^ |  | 594 | 594 |  | ~0 ^***^ |  | 818 | 818 |  | ~0 ^***^ |

The observed (Obs) and expected (Exp) values are for the "+" or reference strand; the values for a dimer and its opposite DNA strands are given to illustrate the orientation on each strand. The expected values are obtained by multiplying the proportion of each dimer involving that base in the genome and the number of mutations of each base. To allow for multiple comparisons, the Benjamini-Hochberg procedure was applied with the false discovery rate set at 1% for the four comparisons per dataset; significant values by this criterion are in bold. Exp, the expected number of mutations; Obs, the observed number of mutations; M, mutated base shown in bold and are underlined; *P*, probability for the Chi-square value for each comparison (the last *P* value in each set is for the Chi-square test of the four obseved values versus the two expected values). *P*~0 is used to indicate *P* values < 10^-10^, and asterisks indicate significant differences as follows: ^***^*P* ≤ 0.001; ^**^*P* ≤ 0.01; ^*^*P* ≤ 0.05.

**Additional Table S4** DNA strand biases of BPSs at the three temperatures.

| Temperature (°C) | Lagging strand template | | | | |  | |  | | Lagging strand template | | | | | |  | |  |
| --- | --- | --- | --- | --- | --- | --- | --- | --- | --- | --- | --- | --- | --- | --- | --- | --- | --- | --- |
|  | C_Obs_ | C_Exp_ | G_Obs_ | G_Exp_ | C/G _Obs_ | | *P* ^†^ | |  | | A_Obs_ | A_Exp_ | T_Obs_ | T_Exp_ | A/T _Obs_ | | *P* ^†^ | |
|  | G:C > A:T transitions | | | | | | |  | | A:T > G:C transitions | | | | | | | |  |
| 25 | 295 | 209 | 137 | 223 | 2.15 | | ~0 ^***^ | |  | | 346 | 267 | 191 | 270 | 1.81 | | ~0 ^***^ | |
| 28 | 285 | 197 | 123 | 211 | 2.32 | | ~0 ^***^ | |  | | 396 | 296 | 198 | 298 | 2.00 | | ~0 ^***^ | |
| 37 | 520 | 363 | 231 | 388 | 2.25 | | ~0 ^***^ | |  | | 573 | 407 | 245 | 411 | 2.34 | | ~0 ^***^ | |

Exp, the expected number of mutations; Obs, the observed number of mutations.

*P* ^†^ is the probability calculated for the Chi-square test of the two observed values versus the two expected values, and asterisks indicate significant differences as follows: ^***^*P* ≤ 0.001; ^**^*P* ≤ 0.01; ^*^*P* ≤ 0.05. The expected ratios for C/G on the lagging-strand template (= the leading template) is 0.94, and the expected for A/T is 0.99.

**Additional Table S5** Ratios of base changes on the transcribed versus the nontranscribed DNA strand.

| Mutational change | 25°C | | |  | 28°C | | |  | 37°C | | |
| --- | --- | --- | --- | --- | --- | --- | --- | --- | --- | --- | --- |
|  | TS/NTS _Obs_ | TS/NTS _Exp_ | *P* |  | TS/NTS _Obs_ | TS/NTS _Exp_ | *P* |  | TS/NTS _Obs_ | TS/NTS _Exp_ | *P* |
| Transitions |  |  |  |  |  |  |  |  |  |  |  |
| A > G | 0.95 | 1.00 | 0.63 |  | 0.84 | 1.00 | 0.06 |  | 0.85 | 1.00 | **0.03**^*^ |
| G > A | 1.15 | 0.90 | **0.01** ^**^ |  | 0.90 | 0.90 | 0.93 |  | 1.08 | 0.90 | **0.01** ^**^ |
| Transversions |  |  |  |  |  |  |  |  |  |  |  |
| A > T | 4.00 | 1.00 | 0.34 |  | 0.67 | 1.00 | 1.00 |  | 1.60 | 1.00 | 0.37 |
| G > T | 1.83 | 0.90 | 0.11 |  | 0.86 | 0.90 | 0.94 |  | 0.73 | 0.90 | 0.67 |
| A > C | 1.30 | 1.00 | 0.21 |  | 0.33 | 1.00 | 1.00 |  | 0.50 | 1.00 | 0.81 |
| G > C | 0.60 | 0.90 | 0.60 |  | 5.00 | 0.90 | 0.48 |  | 2.00 | 0.90 | 0.07 |
| Total |  |  |  |  |  |  |  |  |  |  |  |
| As | 1.02 | 1.00 | 0.77 |  | 0.83 | 1.00 | **0.04**^*^ |  | 0.85 | 1.00 | **0.04** |
| Gs | 1.16 | 0.90 | **0.01** ^**^ |  | 0.92 | 0.90 | 0.78 |  | 1.09 | 0.90 | **0.01** ^**^ |

Exp, the expected ration calculated from the number of each purine on each strand. TS/NTS, the ratio of the number of BPS in the transcribed strand (TS) to the number in the nontranscribed strand (NTS). P, the probability calculated for the Chi-squared test of the observed values on transcribed strand and nontranscribed strand versus the corresponding expected values, if any number is <5, the Yates correction was applied [7]. Asterisks indicate significant differences as follows: ^***^*P* ≤ 0.001; ^**^*P* ≤ 0.01; ^*^*P* ≤ 0.05.

**Additional Table S6** Spectra of indels at three temperatures

| Mutational changes | 25°C | 28°C | 37°C |
| --- | --- | --- | --- |
| Total | 191 ^a^ | 214 ^a^ | 342 ^b^ |
| +1 bp | 100 ^a^ | 121 ^a^ | 215 ^b^ |
| -1 bp | 80 ^a^ | 79 ^a^ | 110 ^a^ |
| + >1 bp | 7 ^a^ | 9 ^a^ | 13 ^a^ |
| - >1 bp | 4 ^a^ | 5 ^a^ | 4 ^a^ |
| +1 A:T | 17 ^a^ | 19 ^a^ | 40 ^a^ |
| -1 A:T | 30 ^ab^ | 48 ^bc^ | 64 ^c^ |
| +1 G:C | 83 ^a^ | 102 ^a^ | 175 ^b^ |
| -1 G:C | 50 ^a^ | 31 ^a^ | 46 ^a^ |
| In run | 189 ^a^ | 212 ^a^ | 341 ^b^ |
| Not in run | 2 ^a^ | 2 ^a^ | 1 ^a^ |

bp, base pair; A run is two or more of the same base pair or repeats of a short base-pair sequence. Data points annotated with different letters had significant difference (*P*_adj_ < 0.05; based on *t* tests, with *P* values from multiple comparisons between temperatures for each data set corrected using the Benjamini-Hochberg procedure).

**Additional References**

1. Lee H, Popodi E, Tang H, Foster PL. Rate and molecular spectrum of spontaneous mutations in the bacterium *Escherichia coli* as determined by whole-genome sequencing. Proc Natl Acad Sci U S A. 2012;109:E2774–83.

2. Foster PL, Lee H, Popodi E, Townes JP, Tang H. Determinants of spontaneous mutation in the bacterium *Escherichia coli* as revealed by whole-genome sequencing. Proc Natl Acad Sci U S A. 2015;112:E5990–9.

3. Wei W, Ning L-W, Ye Y-N, Li S-J, Zhou H-Q, Huang J, et al. SMAL: a resource of spontaneous mutation accumulation lines. Mol Biol Evol. 2014;31:1302–8.

4. Loeb LA, Preston BD. Mutagenesis by apurinic/apyrimidinic sites. Ann Rev Genet. 1986;20:201–30.

5. Gough M, Lederberg S. Methylated bases in the host-modified deoxyribonucleic acid of *Escherichia coli* and bacteriophage lambda. J Bacteriol. 1966;91:1460–8.

6. Coulondre C, Miller JH, Farabaugh PJ, Gilbert W. Molecular basis of base substitution hotspots in *Escherichia coli*. Nature. 1978;274:775–80.

7. Zar JH. Biostatistical Analysis. Englewood Cliffs, NJ: Prentice Hall; 1984.
